# Mobile Alert and Warning in the United States and Japan: Confronting the Challenges of International Harmonization

**DOI:** 10.1007/s13753-021-00380-4

**Published:** 2021-11-15

**Authors:** Hamilton Bean, Ana Maria Cruz, Mika Shimizu, Keri K. Stephens, Matthew McGlone, Sharon Strover

**Affiliations:** 1 Department of Communication, University of Colorado, Denver, CO 80204 USA; 2 Disaster Prevention Research Institute (DPRI), Kyoto University, Kyoto, 611-0011 Japan; 3 Graduate School of Advanced Integrated Studies in Human Survivability, Kyoto University, Kyoto, 606-8501 Japan; 4 Department of Communication Studies, The University of Texas at Austin, Austin, TX 78712 USA; 5 School of Journalism and Media, The University of Texas at Austin, Austin, TX 78712 USA

**Keywords:** Alerts, Disasters, Warning, Japan, Mobile technology, United States

## Abstract

A U.S.-Japan expert workshop on mobile alert and warning was held online 8–10 September 2021. Funded by the Japan Foundation’s Center for Global Partnership (CGP) and responding to the Sendai Framework for Disaster Risk Reduction 2015–2030, the workshop compared U.S. and Japanese mobile alert and warning contexts, systems, policies, and messages to investigate possibilities for international harmonization of mobile device-based early warning. The workshop’s sessions revealed two interrelated issues that repeatedly surfaced among workshop participants: culture and policy. The workshop illuminated several possibilities and problems confronting U.S., Japanese, and global stakeholders as they develop, deploy, and seek to improve the effectiveness of mobile alert and warning systems and messages.

## U.S.-Japan Workshop Background

The U.S.-Japan Expert Workshop: Improving Mobile Public Alert and Warning Globally (hereafter “U.S.-Japan Workshop”) was envisioned in response to warning expert Dr. Dennis Mileti’s (2018) FEMA PrepTalk presentation. In that presentation, Dr. Mileti asked, “If I had to address the men and women in our nation who will issue public alerts and warnings in the future, what would I tell them?” Dr. Mileti responded to his own question with four answers: (1) focus on alert and warning for imminent, rapid onset emergencies; (2) remove delay from the warning system (message issuance, reception, and action initiation); (3) conduct planning, training, and drills for alert and warning; and (4) disseminate alert and warning messages using a mix of new and old technologies. Dr. Mileti underscored that warning messages usually compel people to search for additional and confirming information—not take immediate action. Therefore, in a rapid onset emergency, reducing people’s propensity to “mill” is vital. In his presentation, Dr. Mileti showcased an example of the type of mobile alert and warning message that he believed could be a model for those distributed through the U.S. Wireless Emergency Alerts (WEA) system.

While Dr. Mileti’s remarks focused on the United States, the U.S.-Japan Workshop was intended to explore the international “portability” of Dr. Mileti’s recommendations. The workshop addressed what level of U.S.-Japan mobile alert and warning system and message harmonization was both possible and desirable. The organizers believed that a U.S.-Japan cross-national comparison could shed light on the possibilities and problems confronting mobile alert and warning stakeholders globally. Dr. Mileti’s PrepTalk presentation served as the catalyst of the U.S.-Japan Workshop, but the workshop’s objectives aligned well with “Global Target G” of the Sendai Framework for Disaster Risk Reduction 2015–2030 (UNDRR 2015). Global Target G aims to “substantially increase the availability of and access to multi-hazard early warning systems and disaster risk information and assessments” (UNDRR 2015, p. 12). Availability and access must be accompanied by effectiveness. The Sendai Framework’s Indicators “G-5” objective emphasizes effectiveness when noting that relevant disaster risk information and assessment must be “understandable, usable and relevant” (PreventionWeb 2021). The “G-6” objective also emphasizes effectiveness in seeking to increase the percentage of people who are “protected through pre-emptive evacuation following early warning” (PreventionWeb 2021). The U.S.-Japan Workshop therefore explored how U.S. and Japanese mobile alert and warning stakeholders, especially the public, conceptualizes the meanings of “understandable, usable, and relevant” in ways that promote or impede action, including pre-emptive evacuation. We must stress, however, that these explorations involved only the opinions/perceptions of the workshop participants based on past experiences as citizens and researchers. The observations discussed herein are not always based on published work. The workshop pointed to areas that need to be further investigated systematically.

## International Harmonization: Moving from Speculation to Investigation

In “Early Warning Alerts for Extreme Natural Hazard Events: A Review of Worldwide Practices,” Neußner (2021, p. 2) speculated that the immense variety of early warning schemes globally has resulted in “compromised public awareness and understanding of (early) warning alert messages.” Neußner (2021, p. 2) advocated for “consistent schemes across different hazards and countries, including colour codes (for example yellow, orange, red, for increasing dangers), wordings, pictograms and other features like acoustic signals.” Kelman and Fearnley (2021), however, have argued that local customization is necessary: “the key [to effectiveness] is people, which means starting with people to determine their interests, needs, contributions, and gaps. Any warning should begin with ‘the first mile’ of connecting with the people using it which means tailoring any warning system to them.” Kelman and Fearnley (2021) claimed that “warnings are contextual” and that system “design, implementation, and maintenance must be flexible and dynamic to adjust to changing societies and environments.” International institutions have also speculated about the appropriate level of warning system standardization, with the World Meteorological Organization and International Standards Organization encouraging high levels of standardization, while the International Federation of Red Cross and Red Crescent and United Nations Development Program have emphasized local adaptation (Neußner 2021).

The U.S.-Japan Workshop sought to investigate Neußner’s (2021) vision of “international harmonization” of alert and warning more deeply, assessing how the two counties’ different systems, policies, and messages might illuminate the challenges of standardization of mobile device-based early warning. The workshop organizers believed this comparison could uniquely pinpoint shared possibilities and problems confronting mobile alert and warning stakeholders globally. The workshop involved 25 participants, 8 from the United States and 17 from Japan. The participants included a mix of members of Japanese national and local government organizations, Japanese private sector organizations, and U.S. and Japanese academic institutions, all of whom have a stake in disaster risk reduction. The small number of participants allowed for wide-ranging discussion across the three days of the workshop. Translation support was available throughout the workshop, and the local community session involved consecutive translation support. Although originally conceived as an in-person workshop to be held at Kyoto University’s Disaster Prevention Research Institute (DPRI), COVID-19 restrictions required the workshop organizers to revert to an online format (Zoom). The workshop objectives were to:

1. Explore the development of global guidelines for mobile alert and warning message policy, practice, and research, including the design of mobile public alert and warning messages (handset display characteristics), standard operating procedures for alert originators, and feedback mechanisms.
2. Identify improved empirical methods for measuring and predicting community responses to mobile public alert and warning messages under different environmental, technological, and cultural contexts and disaster scenarios.
3. Assess non-message factors that influence message issuance, reception, action initiation, and resilience including apps and social media, as well as broader issues of mobile infrastructure, public policy, public education, and ethics.

The workshop revealed that the United States and Japan share several mobile alert and warning similarities. Both countries have pioneered the use of mobile devices for alert and warning, with the United States launching the WEA system in 2012, and Japan launching its earthquake early warning (EEW) system in 2007. Both countries are now witnessing increased frequency and intensity of natural hazard-related disasters due to climate change. Both countries have confronted false, inaccurate, and/or insufficient warning messages, although media coverage of such occurrences has been much more pronounced in the United States. Both countries have struggled to improve mobile system geolocation specificity, with the United States now requiring no more than 1/10 of a mile overshoot of WEA messages to avoid “alert fatigue.” Both countries provide people an array of commercial, local, regional, and national push notification platforms, applications, and services. In both countries, a lack of training and drills for both alert originators and local communities has led to non-use or ineffective use and reception of different mobile systems and messages during emergencies. The workshop revealed shared gaps in knowledge and understanding among researchers, disaster management practitioners, and community members. Both in the United States and Japan, experts in mobile alert and warning are typically removed from community lay people, with each group possessing different assumptions about the meaning, role, and influence of mobile alert and warning messages.

Despite these similarities, the U.S.-Japan Workshop uncovered key differences that point to challenges for international harmonization. The remainder of this conference report addresses two interrelated issues that repeatedly surfaced among workshop participants: culture and policy.

### Culture

When discussing culture, it can be easy to slip into generalizations and stereotypes, and we must acknowledge upfront that cultural differences within U.S. and Japanese communities can be as significant and influential as differences across the two countries. Culture always entails both “visible” and “invisible” phenomena (Hall 1959). In both the United States and Japan, community members make personalized risk assessments and protective action decisions in an imperfect information environment. Rarely will mobile alert and warning messages for an imminent threat contain totally complete, accurate, and actionable information. Yet, culture can moderate how community members engage in personalized risk assessment and decision making under conditions of uncertainty. For example, workshop participants noted that studies of U.S. populations have revealed that “restoration of freedom” is an important concept in anticipating how some Americans respond to instructional health messages. Specifically, researchers studying certain U.S. populations often must account for “reactance” (Brehm 1966), that is, the propensity of people to act in ways contrary to a message’s instructional guidance in order to restore their sense of freedom.

By contrast, “perfectionism” surfaced as a concept that helped account for some cases of alert and warning message issuance and response in Japan. In some situations, fear of public criticism or organizational failure might play a role in limiting alert and warning message issuance (a situation that confronts U.S. officials as well). Likewise, workshop participants discussed how some Japanese citizens might be reluctant to take protective action by themselves—or even seek additional and confirming information from others—for fear of appearing hasty or self-serving. One local community volunteer mentioned that it would be helpful to tell Japanese people that it can be acceptable to break the law to ensure one’s survival during a disaster—for example, it would be acceptable to walk across train tracks at non-designated points (which is illegal in Japan) when flood waters are threatening.

Japan’s more collectivist orientation may make reactance a less salient (but not irrelevant) construct there. Some workshop participants noted that community volunteer organizations, and the leaders of those organizations, may play a more influential role in Japan compared to the United States. The leaders of these volunteer organizations are in close contact with municipal officials during an emergency. Whereas mobile alert and warning systems in the United States appear to assume a highly individuated decision-making process, Japanese mobile alert and warning systems are built upon the assumption of a more structured, coordinated, and hierarchical decision-making process. A few workshop participants intimated that some Japanese community members may in fact depend on their volunteer organization’s leaders for guidance and instruction when receiving mobile alert and warning messages. In some cases, this situation could create an unhelpful dependency on officials. When official messages repeatedly fail to accurately predict or respond to actual conditions, recipients learn to discount or ignore them.

These differences point to the issues of “trust,” “expectations,” and “identities,” and the myriad ways those concepts shape persuasion and social influence strategies among officials, citizens, and visitors in both countries. Local community adaptation of alert and warning messages might potentially make messages longer and more detailed (for example, inclusion of local landmarks and physical indicators of danger), but there is a need for concise, quickly understood messages during an imminent emergency. Therefore, workshop participants considered whether and how certain persuasion and social influence models and approaches might be used to explain, predict, and guide collective action, especially agent-based modeling (Hu et al. 2014), social beliefs about internal/external efficacy (Gulevich et al. 2017), agency assignment (McGlone et al. 2012), and loss aversion and framing (Kahneman and Tversky 1979).

Social influence theories have been developed primarily with reference to Western cultural contexts, and it was somewhat unclear how well those theories might account for public response to alert and warning phenomena in Japan. For example, recent research concerning EEW (earthquake early warning) in Japan has demonstrated that there is a significant difference between the responses of “no action” and “I do not care at all” in response to EEW messages. Counterintuitively, Nakayachi and colleagues (2019) showed how despite most Japanese citizens taking “no action” in response to EEW messages, those messages still allowed them to “mentally prepare,” which the citizens themselves found an extremely valuable affordance of the EEW technology. Stakeholders are now in the process of adapting and extending the “Did You Feel It?” (DYFI) program to assess the new EEW ShakeAlert^®^ system in use on the U.S. West Coast, permitting cross-cultural assessments. For example, Ahn and colleagues (2021) conducted surveys with residents of Sendai and Seattle showing that risk perception and perceived effectiveness of EEW were significantly and positively associated with willingness to pay (WTP) for an (improved) EEW application in both regions. However, the researchers also found that having felt fear during previous experiences of an earthquake significantly predicted WTP only in Seattle, and not in Sendai.

Workshop participants found agency assignment a potentially useful concept in the Japanese context. Officials in Hiroshima, Japan have recently experimented with adjustment warning language for typhoon, moving from a focus on imminence and impact to evacuation efficacy and care for one’s neighbors. This “nudge”-oriented language could gain traction in collectivist cultures, where observing the behavior of one’s neighbors may play a significant role in disaster response. Empirical studies of the use of nudge-oriented messaging for disaster response in Japan are just beginning to appear (Ozaki and Nakayachi 2020), but the concept has also sparked questions about the ethicality of highly persuasive (or manipulative) language.

“Fatalism,” “threat level,” and “uncertainty” were concepts that surfaced during the workshop that might productively account for non-compliance with alert and warning messages in both the United States and Japan. Motivating evacuation behavior has been especially difficult in both countries. However, the diversity of urban communities (including the languages spoken within them) in both countries may complicate connections between culture and collective action. Nevertheless, information and communication technologies can link community members together. For example, workshop participants noted various LINE groups in Japan can connect neighbors with each other and are used for disaster preparedness and response. Additionally, one community in Japan, in partnership with academic researchers, has collaborated with officials to develop its own locally customized set of weather warning messages issued through a mobile device application (Takenouchi and Yamori 2020). Similarly, a large community near Austin, Texas created its own text messaging group to communicate during a wildfire preparedness drill (Stephens and Boettner 2020). Culture can shape the nature and extent of social ties and the shared mobile alert and warning practices within a community.

More subtly, culture can shape the types of mobile messages delivered to wireless subscribers in the first place. In the U.S. cultural context, wireless subscribers may receive “Blue Alerts” for threats to law enforcement personnel, “Silver Alerts” for missing elderly community members, “Amber Alerts” for missing or abducted children, as well as other types of messages. The relative infrequency of reported threats to law enforcement, elderly populations, or children in Japan has resulted in no analogous message types being used there. These differences matter in that U.S. wireless subscribers are more likely to receive imminent threat messages within a diverse mobile alert and warning ecology, which necessarily shapes the familiarity, interpretation, and meaning of related messages.

Culture also may play a role in shaping the specific content included in mobile alert and warning messages for different hazards. For example, EEW messages in Japan state “Earthquake! Earthquake! Earthquake!” By contrast, EEW messages, now used on the U.S. West Coast, instruct recipients: “Drop. Cover. Hold on.” Some researchers have argued that Japan has a more developed “preparedness culture” than the United States (Kitagawa 2016). Preparedness culture may be reflected in the lack of explicit instructional guidance included in warning messages in Japan due to people having received training in school and through various public education campaigns. Yet, in Japan, officials are starting to pay more attention to the need for simplified language (“easy Japanese”) to ensure maximum understanding among message recipients.

Despite U.S.-Japan cultural differences, workshop participants explored the question of how certain theories and concepts—including vested interest theory (VI), psychological reactance theory (PRT), language expectancy theory (LET), message salience, self-efficacy, and individual and collective agency—might account for both message construction and recipient response irrespective of cultural context. Research is needed to assess the usefulness of these theories in a cross-national mobile alert and warning context.

### Policy

National-level mobile alert and warning policy can promote or impede community resilience. For example, workshop participants discussed the 3 July 2021 landslide disaster in Atami, Shizuoka Prefecture, Japan that killed 19 people. The Atami case illustrated the challenges of mobile alert and warning policy ambiguity. Specifically, Japan uses a five-level evacuation advisory system developed by the Japan Meteorological Agency (JMA). The JMA recently revised its advisory system based on public feedback that the meanings of certain advisory levels were not well understood. While the JMA provides advisories, mobile alert and warning messages (including those for evacuation) are issued at the discretion of each municipality and/or prefectural government. As reported by Nippon Hoso Kyokai (NHK 2021), in the Atami case, the JMA successfully prompted the Shizuoka prefectural government to issue a Level-4 evacuation warning, but despite multiple contacts between JMA and Atami officials, the municipality only issued a Level-3 warning that instructed people to prepare elderly and vulnerable populations for possible evacuation. Atami officials eventually issued a Level-5 advisory, but only when the landslide was already well underway. Moreover, 85% of surveyed Atami residents reported receiving no email/mobile warning at all. The Atami case illustrated policy gaps among JMA, prefectural governments, and local governments in consistently defining thresholds or triggers for the issuance of mobile alert and warning messages and evacuation instructions. The case underscored the hesitancy of government officials (both U.S. and Japanese) to issue mobile alert and warning messages under conditions of uncertainty.

The Atami case also highlighted how residents in both Japan and the United States may possess valuable disaster information, that is, photos and videos of an imminent threat, but often do not have a way to easily share that information with officials, although some communities in both countries have taken steps to facilitate the two-way flow of disaster information. Nevertheless, the pace and intensity of information sharing over social media and mobile devices create opportunities for the dissemination of misinformation and disinformation. Such misinformation and disinformation can contribute to larger social media narratives that may adversely impact disaster preparedness and response. An adequate approach to collecting and moderating community-generated hazard information—one that can be incorporated into mobile alert and warning systems internationally—has yet to be identified.

It was noted during the workshop that the Atami case in some ways resembled a landmark case in the development of mobile alert and warning policy in the United States. Specifically, in January 2018, a series of mudflows occurred in Santa Barbara County, California, killing 23 people. Both mandatory evacuation orders and voluntary evacuation warnings were issued for residents to leave the area; however, media reporting indicated that Santa Barbara County officials chose not to send a WEA (Wireless Emergency Alert) message to cellphones until destructive mudflows had already begun. The Santa Barbara case, along with multiple instances of mobile alert and warning system non-use during deadly wildfires throughout California, prompted the state legislature to require the Office of Emergency Services to develop and issue statewide guidelines for the use of mobile alert and warning systems in 2019. Unfortunately, it appears that the United States and Japan may also share the experience of witnessing meaningful policy development of mobile alert and warning only in the aftermath of deadly tragedies that disproportionately impact vulnerable groups such as elderly people or access and functional needs populations.

Both cases illustrate the importance of geographic location in relation to mobile alert and warning systems and messages. Specifically, rural communities in Japan typically alert residents of imminent hazards through a community-wide siren and loudspeaker system: *bosai musen*. Verbal warnings and instructions are communicated directly through these systems, as well as via a radio-like information device installed in people’s homes. Outdoor loudspeakers can at times be inaudible, however. In the Atami case, some residents stated that the “tone” of the verbal *musen* announcement lacked the intensity needed to spur protective action. Nevertheless, the use of the *musen* system, coupled with the generally high level of local awareness of disaster risks, may make the use of mobile alert and warning systems less critical in rural communities in Japan. Moreover, in both Japan and the United States, rural communities may not even have the capability to issue mobile alert and warning messages in the first place. Dense urban areas in Japan appear to rarely use *musen* systems, and the higher proportion of non-local residents and international visitors makes the issuance of multilingual mobile alert and warning messages desirable. One international student in an urban area of Japan who received a verbal, *musen*-type warning in Japanese on her mobile device reported that she was greatly alarmed by the message, which she could not understand. However, she was able to use her mobile device to translate the subsequent textual information that she received. Location also matters when considering the channels available to officials and used to communicate disaster information to residents: television, radio, sirens/*musen*, and mobile devices. Location and demographic factors (especially age) will shape the most effective mix of channels. Yet, whether in rural or urban locations, workshop participants agreed that policies that promote community education, training, and drills were essential for promoting better disaster response outcomes.

How to best avoid “alert fatigue” was a question that also arose during the workshop. Mobile alert and warning system policies can promote or impede “over alerting.” One participant noted that in a prior workshop in Japan, participants voiced the idea that alerts should be sent even if they are a “swing and miss” or “end in failure.” Similar to Neußner (2021), this participant suggested that mobile alert and warning messages might gradually intensify, with inclusion of easily understandable levels of caution (for example, green light, yellow light, and red light). Another policy-related question related to the role of hazard type and message issuance. For example, a green/yellow/red light policy approach may make sense for hazards that intensify over time, but as one participant mentioned, there is a tremendous variety of situations in which mobile alert and warning messages may be issued: from earthquake to active shooter to missing elderly persons. Each situation entails unique communication challenges, and the workshop participants agreed that mobile alert and warning “preparedness education,” “literacy,” or “action rules” might be needed to help people make sense of and respond to the multiple (and at times confusing or contradictory) messages they might receive. It may be possible to use mobile devices to communicate these types of educational information. For example, Tokyo officials have installed a type of educational “game” within a mobile device-based preparedness application available to residents. Use of the Personal Tsunami Evacuation Drill app, “Nige-Tore,” can positively impact preparedness outcomes (Yamori and Sugiyama 2020). Of course, mobile alert and warning messages and preparedness activities do not exist in a vacuum and parsing out their unique influence within a given communication ecology will be extremely difficult.

Workshop participants wrestled with the question of the relative importance of message content versus preparedness activity. Expecting mobile alert and warning messages to adequately inform and instruct recipients during an emergency may be misguided: pre-event preparedness activities are needed. Conversely, downplaying or ignoring the content of warning messages may be equally counterproductive. Levels of risk uncertainty may moderate the relative importance of message content versus preparedness activities, but the variability and complexity of disaster situations (including Natech and/or cascading hazards) makes mobile alert and warning policy development and adaptive governance difficult.

## U.S.-Japan Research Collaborations to Improve Mobile Alert and Warning Globally

The U.S.-Japan Workshop revealed that international research collaborations are needed not only to increase the effectiveness of mobile alert and warning to improve risk perception but also to increase protective action compliance. For some officials, ensuring protective action compliance is too difficult and beyond anyone’s control. Yet, researchers are now turning attention to how “terse” messages can address this conceptual gap (Sutton et al. 2021). U.S. and Japanese stakeholders have yet to identify the specific mobile device-based warning and instruction that will compel tangible improvements that advance the Sendai Framework’s G-6 objective, but the occurrence of the U.S.-Japan Workshop signals that important work may be on the horizon. It remains unclear, however, whether or how international “harmonization” should be at the center of that work.

Theoretically speaking, one way to test the possibilities for cross-national system and message harmonization in the future would be to develop an “optimized” alert and warning message that could be used in both U.S. and Japanese evacuation contexts. Optimized and non-optimized messages could then be issued to study participants to track whether any meaningful differences in behavior and safety outcomes occurred between the two groups of recipients (measured via mobile device tracking, self-report, and official after-action reports). If mobile alert and warning stakeholders aim to do more than merely improve the accuracy of risk perception, then such field-based, side-by-side comparisons of warning message types may ultimately be needed (Claeys et al. 2021). However, a field-based approach during an actual emergency could potentially increase risk for some participants and is therefore untenable unless steps could be taken to fully protect research participants. Privacy concerns in both countries could also make device tracking research challenging. Stakeholders will likely have to continue to rely on survey, experimental, and field research to guide policy and practice. The workshop revealed that academic researchers in Japan tend to establish long-term relationships with local communities to promote candid response and exchange, a norm that U.S.-Japan research teams will need to uphold in order to be successful. Developments in the United States are also compelling researchers to now develop more equitable relationships with the communities they study. The workshop participants agreed that centering human factors and systems to serve local communities was critical.

In sum, the U.S.-Japan Workshop revealed some of the possibilities and problems confronting those who seek to “harmonize” mobile alert and warning systems and messages internationally. The National Academies of Sciences, Engineering, and Medicine’s 2018 report, *Emergency Alert and Warning Systems: Current Knowledge and Future Research Directions*, drew primarily from U.S. Department of Homeland Security-funded studies of the WEA system. Although the National Academies report has informed U.S. policy making and practice, to our knowledge, the U.S.-Japan Workshop was the first effort to broadly compare mobile alert and warning systems and messages in the United States and Japan. In several ways, the workshop participants followed Dr. Mileti’s guidance to focus on mobile alert and warning challenges for imminent, rapid onset emergencies and to seek to remove delay from the warning system. In both the Unites States and Japan, planning, training, and drills for mobile alert and warning are needed. In both countries, disseminating alert and warning messages using a mix of new and old technologies is essential. Yet, the workshop also highlighted cultural and policy differences and uncertainties that challenge the desirability of internationally “harmonized” mobile alert and warning.
